# Effects of Dietary Linseed Oil and Propionate Precursors on Ruminal Microbial Community, Composition, and Diversity in Yanbian Yellow Cattle

**DOI:** 10.1371/journal.pone.0126473

**Published:** 2015-05-29

**Authors:** Xiang Z. Li, Byung K. Park, Jong S. Shin, Seong H. Choi, Stephen B. Smith, Chang G. Yan

**Affiliations:** 1 Department of Animal Science, Yanbian University, Yanji, Jilin, 133002, P. R. China; 2 Feed Research Institute, Nonghyup Feed Co. Ltd, Seoul, 134–763, Republic of Korea; 3 Department of Animal Life Science, Kangwon National University, Chunchoen, 200–701, Republic of Korea; 4 Department of Animal Science, Chungbuk National University, Cheongju, Chungbuk, 361–763, Korea; 5 Department of Animal Science, Texas A & M University, College Station, 77843–2471, United States of America; Agriculture and Agri-Food Canada, CANADA

## Abstract

The rumen microbial ecosystem is a complex system where rumen fermentation processes involve interactions among microorganisms. There are important relationships between diet and the ruminal bacterial composition. Thus, we investigated the ruminal fermentation characteristics and compared ruminal bacterial communities using tag amplicon pyrosequencing analysis in Yanbian yellow steers, which were fed linseed oil (LO) and propionate precursors. We used eight ruminally cannulated Yanbian yellow steers (510 ± 5.8 kg) in a replicated 4 × 4 Latin square design with four dietary treatments. Steers were fed a basal diet that comprised 80% concentrate and 20% rice straw (DM basis, CON). The CON diet was supplemented with LO at 4%. The LO diet was also supplemented with 2% dl-malate or 2% fumarate as ruminal precursors of propionate. Dietary supplementation with LO and propionate precursors increased ruminal pH, total volatile fatty acid concentrations, and the molar proportion of propionate. The most abundant bacterial operational taxonomic units in the rumen were related to dietary treatments. Bacteroidetes dominated the ruminal bacterial community and the genus *Prevotella* was highly represented when steers were fed LO plus propionate precursors. However, with the CON and LO diet plus malate or fumarate, Firmicutes was the most abundant phylum and the genus *Ruminococcus* was predominant. In summary, supplementing the diets of ruminants with a moderate level of LO plus propionate precursors modified the ruminal fermentation pattern. The most positive responses to LO and propionate precursors supplementation were in the phyla Bacteriodetes and Firmicutes, and in the genus *Ruminococcus* and *Prevotella*. Thus, diets containing LO plus malate or fumarate have significant effects on the composition of the rumen microbial community.

## Introduction

The rumen microbial ecosystem is a complex system where rumen fermentation involves highly complex interactions among microorganisms [1]. Rumen microflora are known to be highly responsive to changes in diet [2, 3], and the composition and type of diet are critical factors that affect rumen microbial activity and rumen function [4]. Therefore, a general understanding of complex ruminal microbial populations, their interactions, and their response to different diets is important.

Dietary lipid and propionate precursors may affect the rumen microbial profile and the composition of fermentation endproducts [5]. Based on *in vitro* studies, certain plant oils have been shown to increase propionate and decrease lactate and methane [5, 6], and the rumen microbial diversity responded clearly to the biohydrogenation process involved in dietary unsaturated fatty acids metabolism. Dicarboxylic acids such as fumarate and malate, which are propionate precursors in the pathway from succinate to propionate [7], act as H_2_ acceptors [8]. Dicarboxylic acids can be used by rumen microorganisms to produce propionate, decrease methane production, and increase total volatile fatty acids (VFA) [3]. A previous *in vitro* study [6] found that α-linolenic acid (C18:3n-3; ALA) in association with malate or fumarate increased total VFA production and propionate proportion and reduced methane generation by rumen microbes. A preliminary *in vivo* study showed that dietary supplementation with linseed oil (LO; an oil that is enriched with ALA) plus malate or fumarate increased dry matter (DM) and neutral detergent fiber (NDF) digestibility compared with LO supplementation only (data not reported). Malate and fumarate act as alternative electron sinks and they may compete with methane generation and the biohydrogenation of ALA for the utilization of metabolic hydrogen, thereby affecting the fermentation characteristics and metabolic intermediates produced from ALA. We hypothesized that dietary LO and propionate precursors have differential effects on bacterial populations, possibly by stimulating the growth of major ruminal bacteria, thereby affecting mixed microorganism ruminal fermentation.

Our knowledge of the bacterial diversity in the rumen has increased with the development of novel molecular microbiology techniques [9–12]. In particular, pyrosequencing is a high-throughput analytical method that can be used to generate very large amounts of DNA reads through a massively parallel sequencing-by-synthesis approach [13]. Using the high-throughput pyrosequencing method, Jami and Mizrahi [14] investigated the rumen microbiota composition and identified similarities and differences among the rumen bacteria obtained from individual lactating cows fed the same diet. Therefore, the present study aimed to identify the composition of the overall bacterial community in the rumen ecosystem and to determine the effects of dietary LO and propionate precursors (malate and fumarate) on the fermentation characteristics in Yanbian yellow steers, where we utilized 454 tag amplicon pyrosequencing analysis.

## Results

### Rumen fermentation characteristics

The LO-M diet (2% of the concentrate in the LO diet supplemented with dl-malate), and the LO-F diet (2% of the concentrate in the LO diet supplemented with fumarate) increased ruminal pH at 3 h (*P* < 0.042) and 6 h (*P* < 0.021) after feeding compared to the CON diet and the LO diet (Table 1). Ammonia-N concentration in the rumen fluid was not affected by dietary supplements (*P* > 0.362). The LO, LO-M, and LO-F diets decreased total VFA concentrations in rumen fluid at 3 h (*P* < 0.037) and 6 h (*P* < 0.046) compared with the CON diet. The LO, LO-M, and LO-F diets decreased the concentration of acetate (C2) at 6 h (*P* < 0.026) after feeding and increased the concentration of propionate (C3) at 3 h (*P* < 0.015) and 6 h (*P* < 0.045) after feeding compared to the CON diet. The C2/C3 ratios were lower with the LO, LO-M, and LO-F diets at 3 h (*P* < 0.012) and 6 h (*P* < 0.034) after feeding compared with the CON diet. Furthermore, the concentration of butyrate was lower with the LO-M and LO-F diets at 3 h (*P* < 0.027) and 6 h (*P* < 0.037) after feeding compared to results with the CON and LO diets.

**Table 1:** Effects of linseed oil and propionate precursors on the rumen fermentation characteristics in Yanbian yellow steers.

Parameter	Treatment^1^				SEM	*Pr*<F
	CON	LO	LO-M	LO-F		
	3 h post-feeding					
pH	6.50^b^	6.58^b^	6.72^a^	6.74^a^	0.11	0.042
NH_3_-N (mg/100 mL)	22.44	18.94	21.16	18.26	3.12	0.362
Total VFA (mmole/100 mL)	78.77^a^	66.65^b^	69.04^b^	69.72^b^	3.44	0.037
Acetate (C2, mmole/100 mL)	48.55^a^	36.34^b^	43.72^a^ ^b^	42.18^a^ ^b^	2.63	0.031
Propionate (C3, mmole/100 mL)	14.88^b^	16.43^a^	17.91^a^	16.68^a^	0.81	0.015
Butyrate (mmole/100 mL)	12.19^a^	11.58^a^	9.08^b^	9.07^b^	3.12	0.027
C2/C3	3.26^a^	2.21^b^	2.44^b^	2.53^b^	0.18	0.012
	6 h post-feeding					
pH	6.48^b^	6.62^b^	6.84^a^	6.83^a^	0.14	0.021
NH_3_-N (mg/100 mL)	18.22	18.76	22.49	18.56	3.43	0.518
Total VFA (mmole/100 mL)	77.70^a^	66.36^b^	68.82^b^	69.55^b^	4.25	0.046
Acetate (C2, mmole/100 mL)	47.81^a^	37.12^b^	41.24^b^	38.39^b^	2.96	0.026
Propionate (C3, mmole/100 mL)	13.51^b^	15.14^a^	15.98^a^	15.72^a^	0.64	0.048
Butyrate (mmole/100 mL)	11.50^a^	11.21^a^	8.86^b^	8.27^b^	1.37	0.037
C2/C3	3.54^a^	2.45^b^	2.58^b^	2.61^b^	0.24	0.034

^a,b^ Within a row, values without the same superscript are significantly different (*P* < 0.05).

^1^ CON, steers were fed the basal diet only; LO, steers were fed the CON diet supplemented with linseed oil (4% of diet DM); LO-M, steers were fed the CON diet supplemented with linseed oil (4% of diet DM) and malate (2% of diet DM); LO-F, steers were fed the CON diet supplemented with linseed oil (4% of diet DM) and fumarate (2% of diet DM). VFA, volatile fatty acids.

### Taxonomic assignment

**Fig 1:**
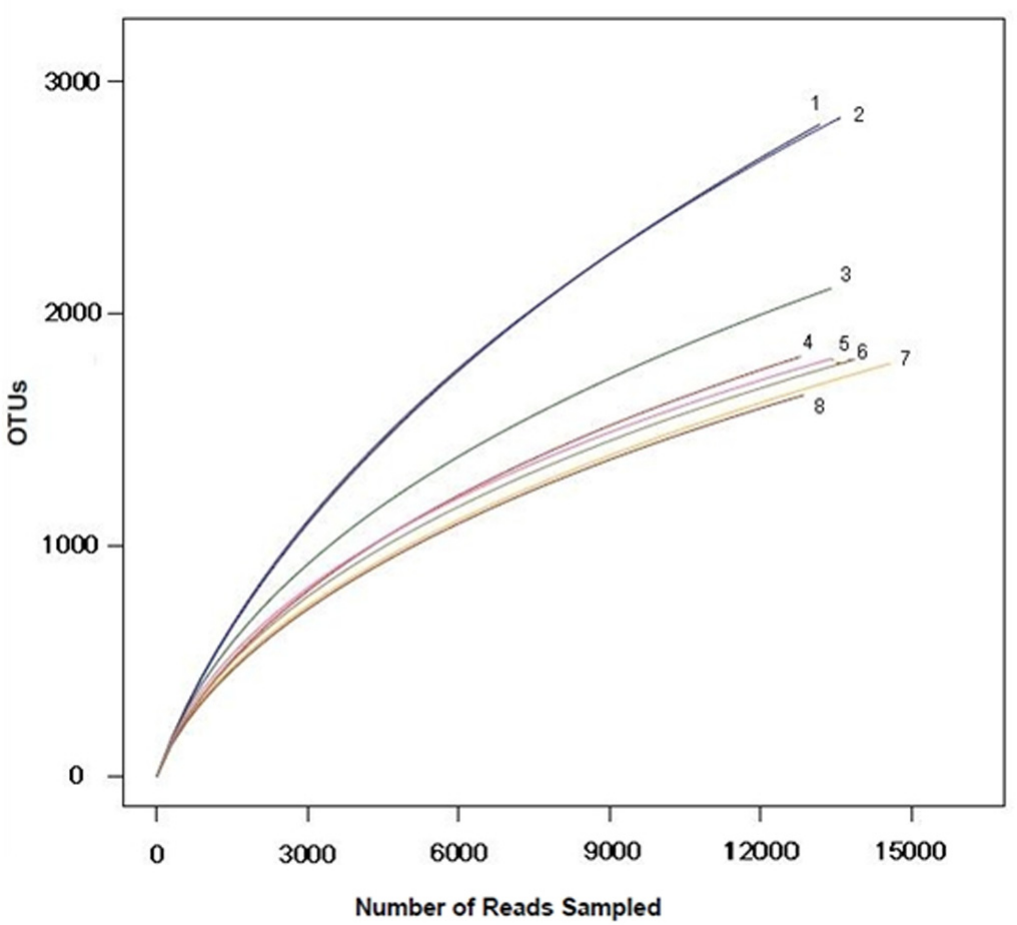
Rarefaction curves of bacterial species richness to assess the average OTU contents. Rarefaction curves of OTUs clustered with ≥97% sequence identity among different samples. 1, CON (3 h); 2, CON (6 h); 3, LO (6 h); 4, LO (3 h); 5, LO-F (6 h); 6, LO-F (3 h); 7, LO-M (3h); 8, LO-M (6 h).

Fig 1 shows the number of operational taxonomic units (OTUs) recovered as a function of the number of sequence reads. In total, 148,000 valid reads and 6,571 OTUs were obtained from the eight samples using 454 pyrosequencing analysis. These sequences/OTUs were assigned to 21 different phyla or groups and each of the eight communities contained >12,000 reads. The number of reads was sufficient to cover most of the biodiversity present. Good’s coverage estimates showed that 94–98% of the species were obtained in all samples.

**Fig 2:**
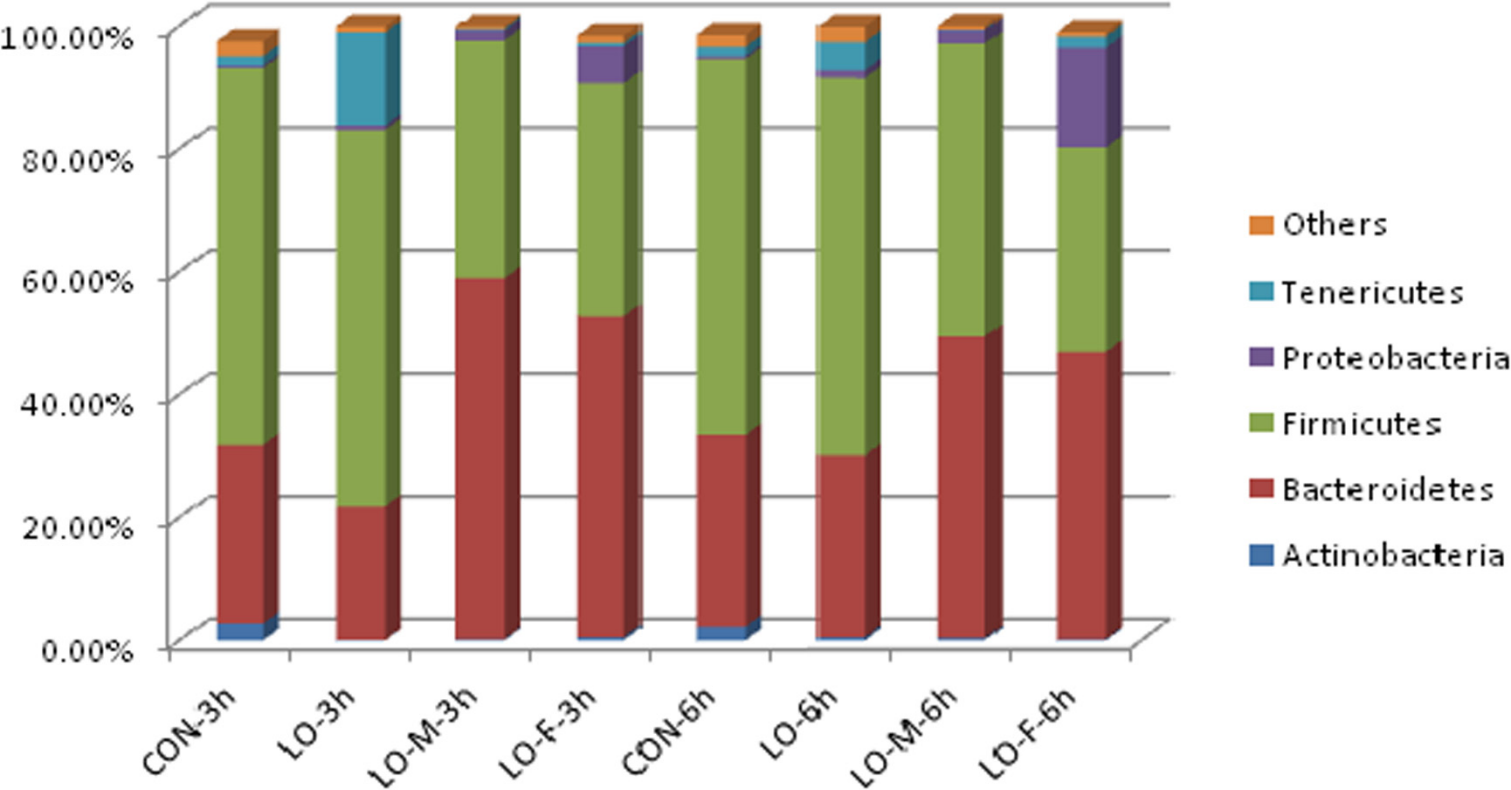
Bacterial compositions of different communities with various experimental diets at 3 h and 6 h after feeding. Relative read abundance of various bacterial phyla in the different communities. CON, steers were fed the basal diet only; LO, fed the CON diet supplemented with linseed oil (4% of diet DM); LO-M, fed the CON diet supplemented with linseed oil (4% of diet DM) and malate (2% of diet DM); LO-F, fed the CON diet supplemented with linseed oil (4% of diet DM) and fumarate (2% of diet DM).

The ruminal bacterial compositions of the different communities are shown in Fig 2. All of the sequences were classified to phylum and genus using the mothur program with the default settings (http://www.mothur.org/wik/Main_Page). Different phyla were identified in the CON, LO, LO-M, and LO-F samples at 3 h and 6 h after feeding. The eight libraries had highly dissimilar 16S rRNA profiles, even at the phylum level. The taxonomic assignment showed that the dominant ruminal bacterial phyla were Bacteroidetes and Firmicutes in all treatment groups, which accounted for 90.45% (CON), 83.03% (LO), 97.46% (LO-M), and 90.21% (LO-F) of total reads at 3 h after feeding, and these two phyla accounted for 92.44% (CON), 91.04% (LO), 96.77% (LO-M), and 80.03% (LO-F) of the total reads at 6 h after feeding.

The following results are reported as bacterial phyla with percentages for 3 h and 6 h, respectively, in parentheses. At 3 h and 6 h after feeding, the CON library had relatively high diversity, which was dominated by Firmicutes (61.33%, 61.22%), Bacteroidetes (29.12%, 29.63%), and Tenericutes (1.55%, 1.56%,). The LO library was dominated by Firmicutes (61.27%, 61.41%), Bacteroidetes (21.76%, 29.63%), and Tenericutes (15.14%, 4.59%). The LO-M library was dominated by Bacteroidetes (58.73%, 49.11%), Firmicutes (38.73%, 47.66%), and Proteobacteria (1.59%, 2.17%). The LO-F library was dominated by Bacteroidetes (52.25%, 46.74%), Firmicutes (37.93%, 33.29%), and Proteobacteria (5.96%, 16.25%).

**Fig 3:**
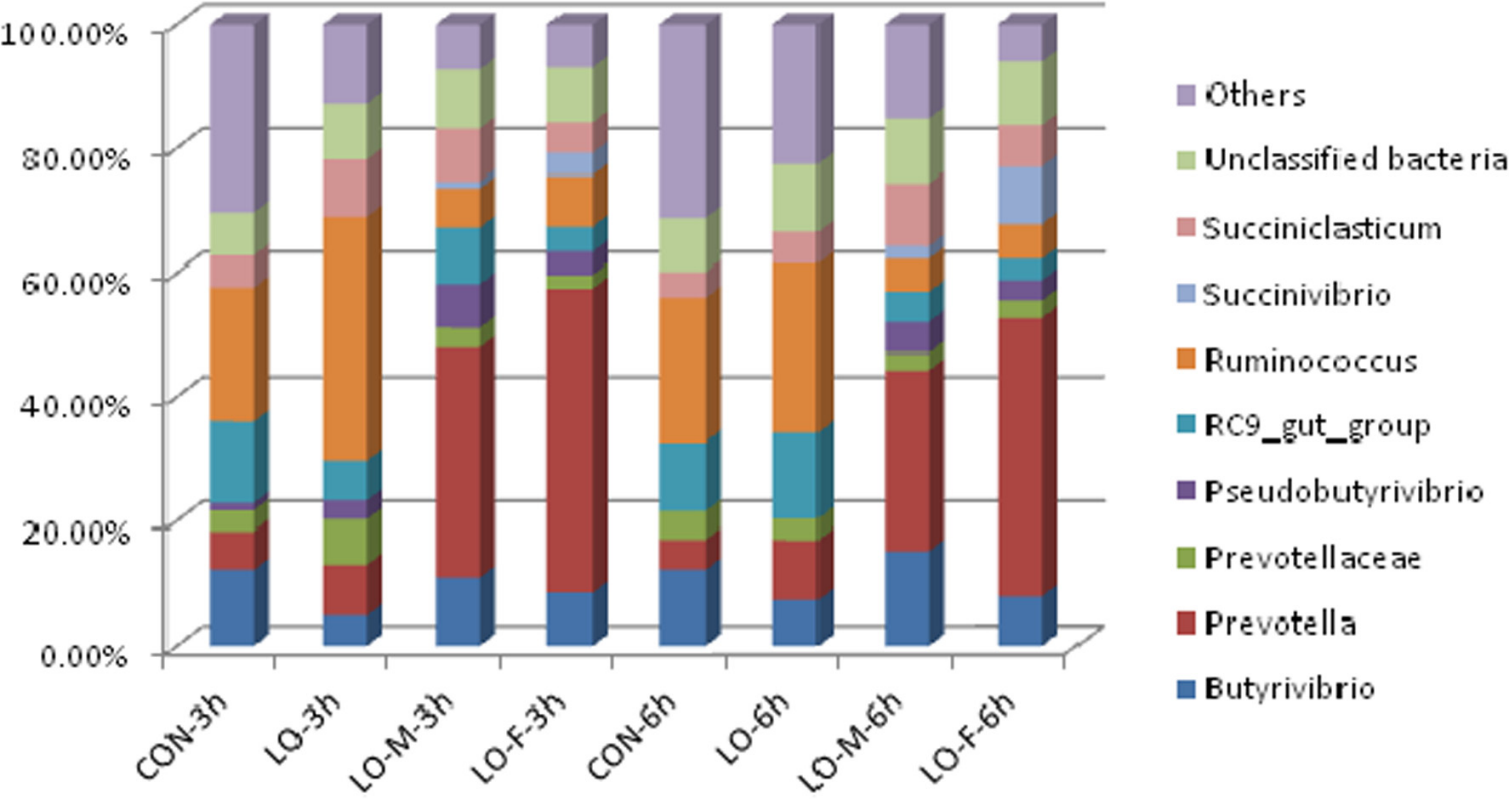
Bacterial taxa determined by pyrosequencing based on the 16S rDNA with various experimental diets at 3 h and 6 h after feeding. Relative read abundance of different bacteria genera in different communities. Taxa that represented <5% were assigned as “Others.” Sequences that could not be classified into any known group were assigned to “Unclassified bacteria.” CON, steers were fed a basal diet only; LO, fed the CON diet supplemented with linseed oil (4% of diet DM); LO-M, steers were fed the CON diet supplemented with linseed oil (4% of diet DM) and malate (2% of diet DM); LO-F, steers were fed the CON diet supplemented with linseed oil (4% of diet DM) and fumarate (2% of diet DM).

The most abundant OTUs were identified in the different samples to determine the dominant bacteria genus (Fig 3). The most abundant OTUs associated with the CON library at 3 h after feeding were sequences related to *Ruminococcus* (21.64%), RC9-gut-group (13.04%), *Butyrivibrio* (12.19%), and *Prevotella* (6.26%). The LO library at 3 h after feeding was dominated by sequences related to *Ruminococcus* (39.03%), *Succiniclasticum* (9.14%) and *Prevotella* (8.29%). The LO-M library at 3 h after feeding was dominated by sequences related to *Prevotella* (36.95%), *Butyrivibrio* (10.92%), RC9-gut-group (9.05%), *Succiniclasticum* (8.48%), and *Pseudobutyrivibrio* (7.09%). The LO-F library at 3 h after feeding was dominated by sequences related to *Prevotella* (49.03%), *Butyrivibrio* (8.45%), *Ruminococcus* (8.22%), and *Succiniclasticum* (5.02%).

At 6 h after feeding, the most abundant OTUs associated with the CON library were sequences related to *Ruminococcus* (23.24%), *Butyrivibrio* (12.20%), RC9-gut-group (11.11%), and *Prevotella* (4.91%) (Fig 3). The LO libraries were dominated by sequences related to *Ruminococcus* (27.27%), RC9-gut-group (13.76%), *Prevotella* (9.79%), and *Butyrivibrio* (7.24%). The LO-M library was dominated numerically by sequences related to *Prevotella* (29.12%), *Butyrivibrio* (15.24%), *Succiniclasticum* (10.18%), *Ruminococcus* (5.21%), and *Pseudobutyrivibrio* (5.06%). The LO-F library was dominated by sequences related to *Prevotella* (44.83%), *Butyrivibrio* (9.19%), *Succinivibrio* (7.87%), and *Succiniclasticum* (6.76%).

### Bacterial similarity

**Fig 4:**
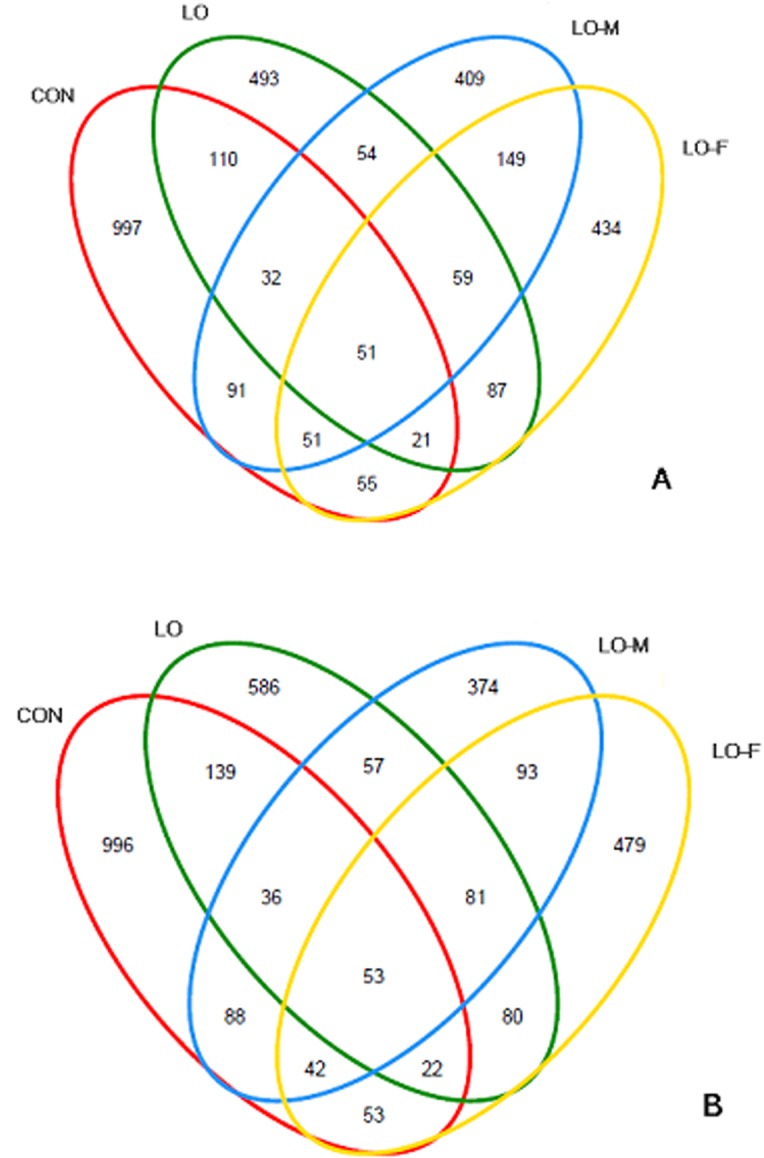
Shared OTUs analysis for different libraries. Venn diagram showing the unique and shared OTUs at distances of 3% in the different libraries: (A) for the CON, LO, LO-M, and LO-F libraries at 3 h after feeding; and (B) for the CON, LO, LO-M, and LO-F libraries at 6 h post-feeding.

The bacterial species present in multiple communities were analyzed by using a Venn diagram to compare the detailed relationships among the communities (Fig 4). The Venn diagram identified the number of OTUs shared between and unique to each of the respective diets. The CON, LO, LO-M, and LO-F libraries shared 51 and 53 OTUs at 3 h and 6 h after feeding, respectively. The number of shared OTUs at 3 h that overlapped with the 6 h group was 849. At 3 h after feeding, the total number of OTUs in the CON library (1,408) was higher than that in each of the LO (907), LO-M (896), and LO-F (907) libraries (Fig 4A). At 6 h after feeding, similar results were obtained for the CON (1,429), LO (1,054), LO-M (824), and LO-F (903) libraries (Fig 4B). Furthermore, the LO-M library had the lowest number of OTUs at 3 h (896) and 6 h (824) after feeding. The number of shared OTUs in the LO, LO-M, and LO-F libraries were 110 and 134 at 3 h and 6 h after feeding, respectively. This was higher than the number of shared OTUs in the CON, LO, and LO-M libraries (83 OTU at 3 h and 89 OTU at 6 h) as well as the CON, LO, and LO-F libraries (72 OTU at 3 h and 75 OTU at 6 h). The LO-M and LO-F libraries shared the highest number of OTUs at 3 h (310) and 6 h (269) after feeding, where the most abundant OTUs shared by these two communities were *Prevotella* and *Butyrivibrio*. Furthermore, the *Succinivibrio* group was found only in the LO-M and LO-F libraries.

**Fig 5:**
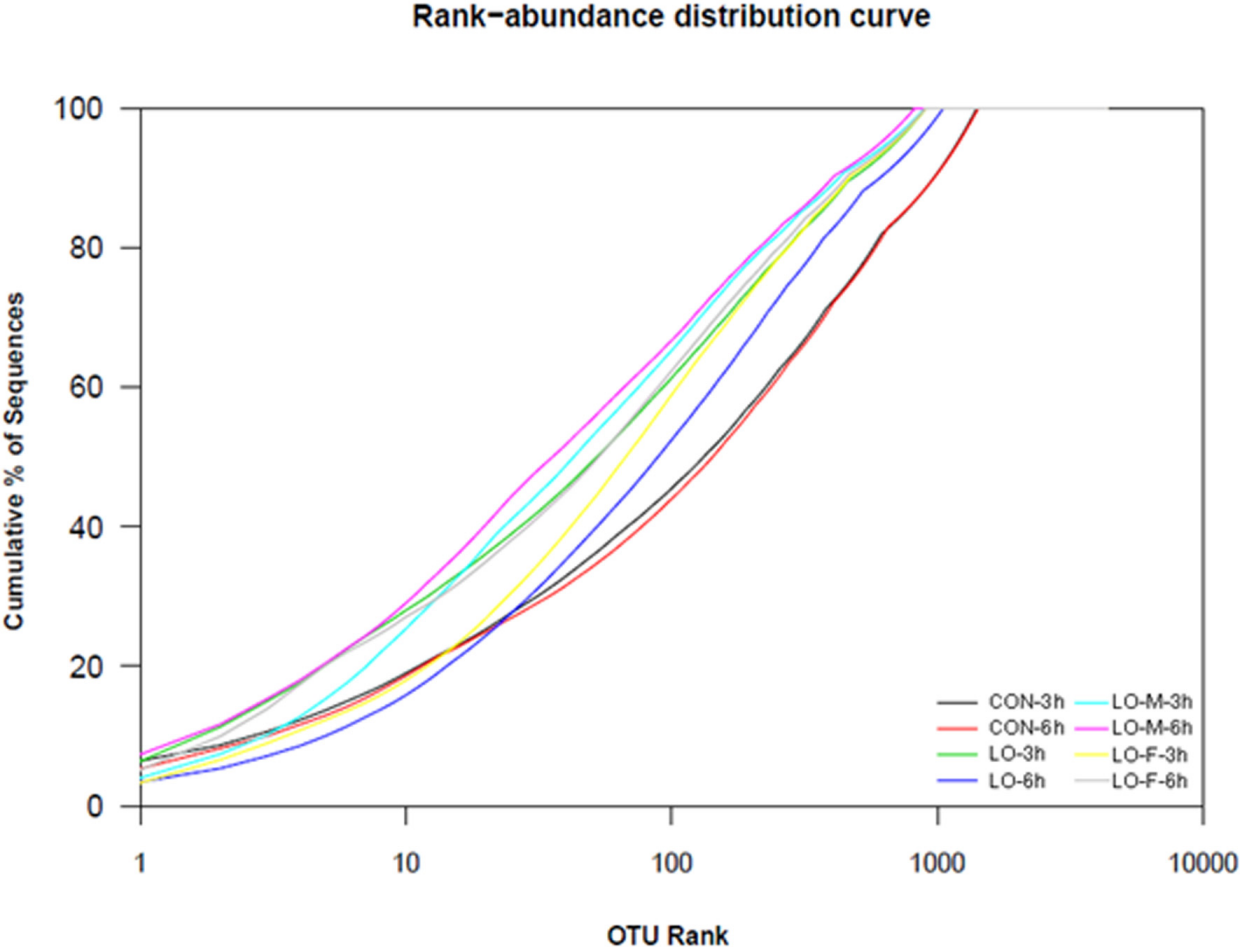
Rank abundance analysis of different bacterial community groups. CON, steers were fed a basal diet only; LO, steers were fed the CON diet supplemented with linseed oil (4% of diet DM); LO-M, steers were fed the CON diet supplemented with linseed oil (4% of diet DM) and malate (2% of diet DM); LO-F, steers were fed the CON diet supplemented with linseed oil (4% of diet DM) and fumarate (2% of diet DM).

**Fig 6:**
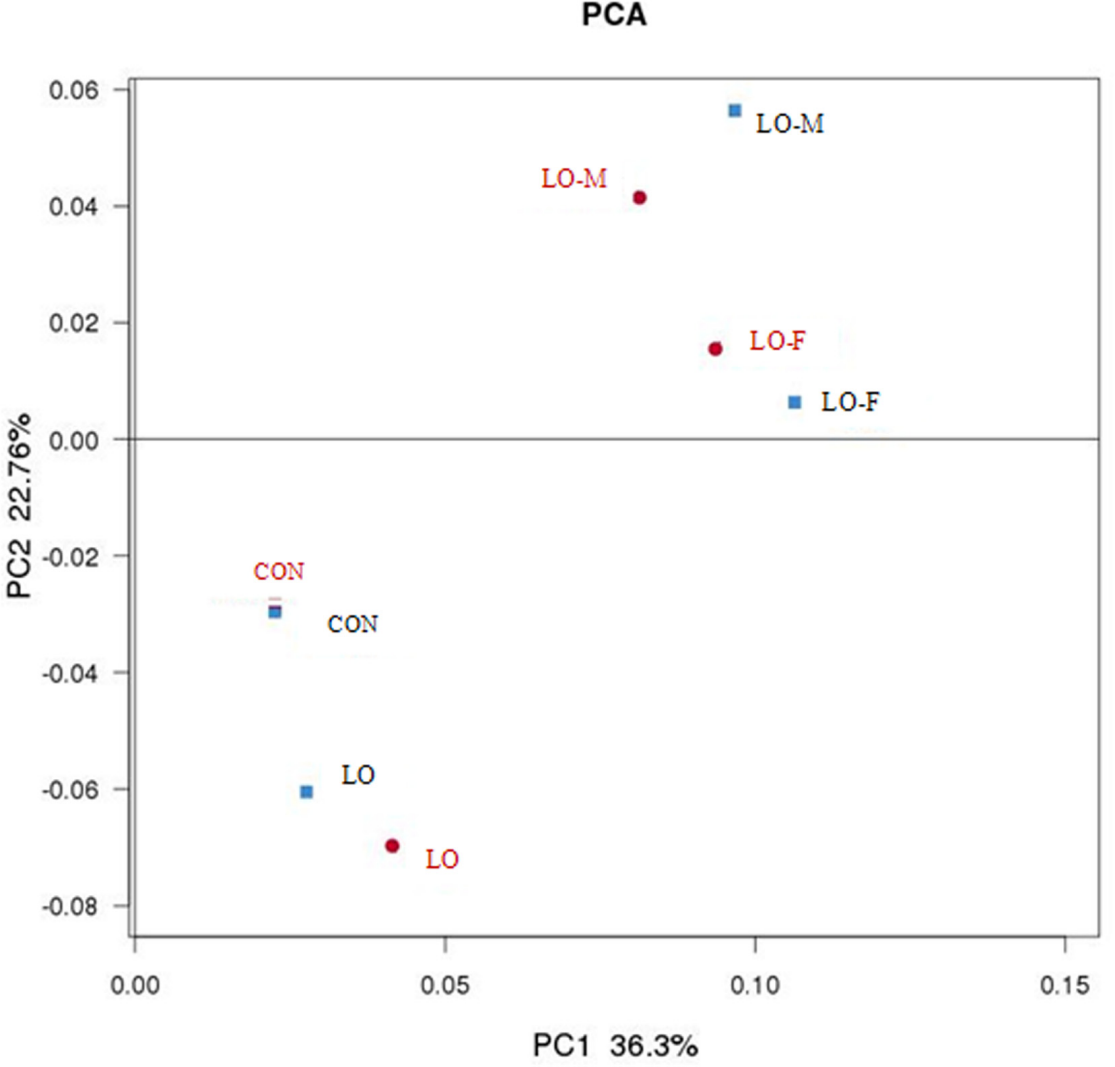
Principal components analysis of samples based on the compositions of the microbial communities. CON, steers were fed a basal diet only; LO, steers were fed the CON diet supplemented with linseed oil (4% of diet DM); LO-M, steers were fed the CON diet supplemented with linseed oil (4% of diet DM) and malate (2% of diet DM); LO-F, steers were fed the CON diet supplemented with linseed oil (4% of diet DM) and fumarate (2% of diet DM). Red dot: sampling at 3 h after feeding. Blue dot: sampling at 6 h after feeding.

**Fig 7:**
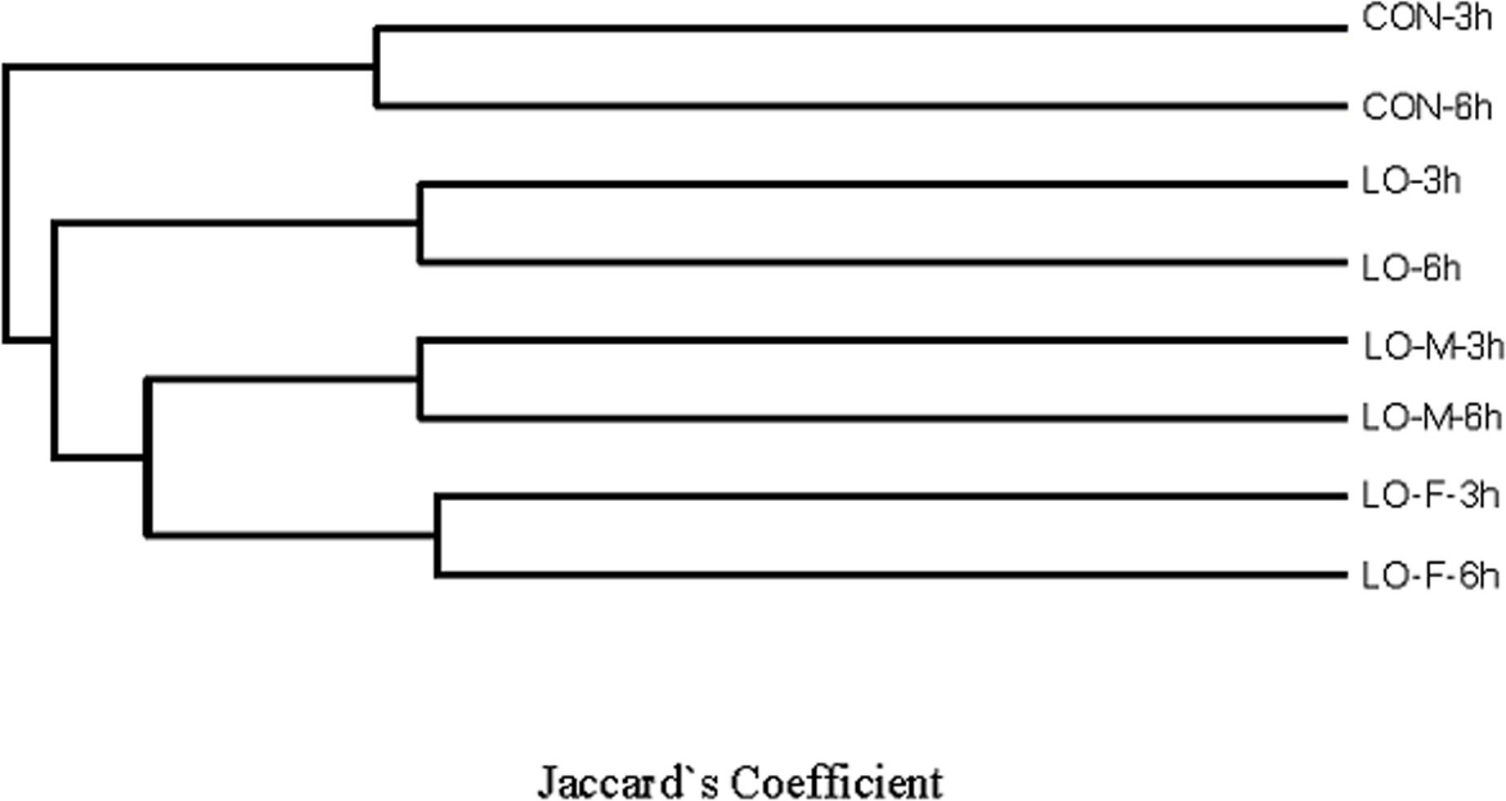
Bacteria community dissimilarity analysis (97% similarity level). Comparison of the similarity of bacteria communities, where we used the “Jaccard’s coefficient using richness estimator” to determine the dissimilarity (1-similarity) between samples. CON, steers were fed a basal diet only; LO, steers were fed the CON diet supplemented with linseed oil (4% of diet DM); LO-M, steers were fed the CON diet supplemented with linseed oil (4% of diet DM) and malate (2% of diet DM); LO-F, steers were fed the CON diet supplemented with linseed oil (4% of diet DM) and fumarate (2% of diet DM).

Rank abundance distribution curves of the OTUs within each category of the Venn diagram (Fig 4) were ranked according to their abundance in the corresponding combined OTU sequence dataset (Fig 5). To assess whether the microbial communities were significantly different, we performed principal components analysis (PCA) and created a phylogenetic tree based on the weighted UniFrac distance (data not shown). The PCA score plot showed that the LO-M and LO-F samples were grouped toward the upper region of the graph along PC2, which accounted for 22.7% of the total variation. The CON samples were closely related to the LO samples, but these samples differed from the LO-M and LO-F samples. Overall, the two PCA axes explained 59.0% of the variation between the different communities (Fig 6). Figs 6 and 7 illustrate the relatively dissimilar diversity of the bacterial communities in the different libraries at 3 h and 6 h after feeding. The LO-M and LO-F communities appeared to be distinct from the CON and LO communities at both 3 h and 6 h after feeding. A hierarchically clustered heat map analysis based on the bacterial community profiles at the family level showed that LO-M and LO-F grouped together first and they were then clustered with the LO and CON samples in that order (Fig 7).

## Discussion

Rumen bacteria are highly responsive to changes in diet [2, 3], which may result in alterations to microbial metabolic activities and microbial communities. In the current study, rumen microbial communities were analyzed to determine the total number of bacterial OTUs and taxa present, and to characterize the similarity of bacterial communities across a relatively large array of different steer rumens. We also identified the universally distributed community using the Roche/454 next-generation Titanium sequencing platform.

Malate and fumarate increased the rumen pH [3, 15], which may have stimulated lactate utilization by the predominant ruminal bacteria [16], thereby decreasing the concentration of lactate [17]. Khampa et al. [18] demonstrated that the supplementation of food concentrates with different levels of sodium dl-malate significantly increased ruminal pH and maintained a more stable pH in dairy steers. Li et al. [19] showed that the addition of linoleic acid plus malate or fumarate to *in vitro* cultures increased pH, total VFA concentrations, and the concentration of propionate. Other studies reported that diets containing malate and fumarate increased lactate metabolism, resulting in an increased ruminal pH in steers [20]. In the present study, the LO-M and LO-F diets increased the concentration of propionate, which suggests that malate and fumarate were metabolized to propionate by rumen bacteria. Similar results were reported based on *in vitro* studies [8]. LO supplementation also increased the proportion of propionate in the current study. Demeyer and Van Nevel [21] reported that the lipid-induced reduction of rumen methanogenesis was accompanied by a shift to increased propionate production.

The increased ruminal pH in this study may be attributable to *Prevotella*, which represented the most abundant OTUs associated with the LO, LO-M, and LO-F supplementation libraries. Leaflet [22] reported that cows treated with *Prevotella* exhibited significantly lower ruminal lactate concentrations and higher total VFA concentrations throughout the experimental period. In this study, the rumen bacterial compositions at 3 h and 6 h after feeding were clearly dominated by members of the genus *Prevotella* in the LO-M and LO-F libraries. Further analysis of this core community at the species level (>97%) showed that 1,411 OTUs were shared among all samples, where *Prevotella* accounted for 1,214 OTUs (data not shown). However, the genus *Ruminococcus* predominated in the CON and LO libraries.

There are important relationships between diet and gastrointestinal bacterial populations and diversity [4, 23]. Previous surveys of rumen microbiota suggest that Firmicutes and Bacteroidetes are the numerically dominant phyla. Indeed, Tajima et al. [24] reported that 52.4% of the clones identified in the rumen liquor of Holstein cows fed a hay diet belonged to Firmicutes and 38.1% to Bacteroidetes. Edwards et al. [25] reported that 54% of rumen bacteria belonged to Firmicutes and 40% to Bacteroidetes. Using tag-encoded amplicon pyrosequencing analysis, Jami and Mizrahi [14] reported that the coverage composition of the rumen bacterial community mainly comprised Firmicutes (43%) and Bacteroidetes (50%). The results were similar in our study, where Firmicutes and Bacteroidetes were the predominant phyla in all libraries.

Interestingly, the relative ratios of Firmicutes and Bacteroidetes varied considerably among the community compositions of the different diet libraries. In the CON and LO libraries, Firmicutes was the most abundant phylum, followed by Bacteroidetes. The ratios of Firmicutes relative to Bacteroidetes were 2.10 (CON) and 2.82 (LO) at 3 h after feeding, and 1.96 (CON) and 2.07 (LO) at 6 h after feeding. The ratios of Firmicutes relative to Bacteroidetes were 0.66 (LO-M) and 0.73 (LO-F) at 3 h after feeding, and 0.97 (CON) and 0.71 (LO) at 6 h after feeding. We propose that this result was caused by the propionate precursors in the LO-M and LO-F diets. Malate and fumarate are intermediates in the pathway from succinate to propionate and therefore they may stimulate ruminal Proteobacteria populations. In the present study, Proteobacteria were another prevalent member of the rumen bacterial communities with the LO-M and LO-F diets but not with the CON and LO diets. Previous research demonstrated that *Prevotella ruminicola* and other bacteria such as *Selenomonas ruminantium*, *Veillonella alcalescens*, and others [26–28] converted succinate to propionate and CO_2_ by decarboxylation.

Some of the shared genera were highly abundant in the overall rumen bacterial communities with all treatments, such as Ruminococcus and *Prevotella*. However, most of the shared genera varied in abundance among the different diets. With the CON and LO diets, *Ruminococcus* (the Firmicutes group) was the dominant genus. However, *Prevotella* (the Bacteroidetes group) was highly represented in the shared microbial community and it was the most abundant genus when the LO-M and LO-F diets were fed. Most of the Firmicutes sequences belonged to the order Clostridiales, in which Lachnospiraceae and Ruminococcaceae were the most highly represented families. These two families are degraders of pectin and cellulose, respectively, and they are important for the gastrointestinal fermentation of dietary fiber [29]. The genus *Prevotella* belongs to the phylum Bacteroidetes, which contains carbohydrate-fermenting and H_2_-producing bacteria that are implicated in energy production [30]. *Prevotella ruminicola* is known to use extracellular H_2_ to convert fumarate to succinate [31]. Therefore, the combination of the LO diet with malate or fumarate caused the predominant bacteria to differ. In addition, the genus *Succinivibrio* (Proteobacteria) was represented in the shared LO-M and LO-F microbial community, so these dietary treatments affected the most active bacterial genera and populations. The genus *Prevotella* was highly abundant after feeding the LO-M and LO-F diets, which has interesting implications for the study of rumen ecology. *Butyrivibrio* is known to be involved in the biohydrogenation of fatty acids [32]. Kim et al. [33] reported that linoleic acid inhibited the growth of *Butyrivibrio*, and a higher *Butyrivibrio* population was detected after supplementation with LO, LO-M, and LO-F. The metabolism of unsaturated fatty acids in LO may have stressed or inhibited the activity of the *Butyrivibrio* population [34]. *Succinivibrio* species are often the predominant isolates from the rumens of cattle fed high-starch diets [35,36] and they are related to propionate production from succinate [28].

In summary, feeding ruminants a diet supplemented with a moderate level of LO and propionate precursors significantly modified the ruminal fermentation pattern. The strongest responses to the supplemented diets occurred in the phyla of Bacteriodetes and Firmicutes, and in the genus *Ruminococcus* and *Prevotella*. This indicates that LO and propionate precursors have significant effects on the composition of the rumen microbe community. These observations substantially increase our understanding of the rumen microbial ecosystem and suggest new means to modify ruminal metabolism.

## Materials and Methods

### Animals and diets

All of the experimental procedures used in this study were approved by the Animal Care and Use Committee of Yanbian University, China. The experiment was conducted using eight ruminally cannulated Yanbian Yellow steers (510 ± 5.8 kg) in a replicated 4 × 4 Latin square design with four dietary treatments. Each 25-day experimental period comprised a 21-day period of adaptation to treatment and a 4-day sampling period. The steers were housed in individual pens with free access to water. The steers were fed a basal diet that contained 80% concentrate (Cofeed Ltd, Changchun, China; crude protein (CP): 17%, NDF: 20%) and 20% rice straw (DM basis, CON) (Table 2; AOAC [37]). The CON diet was supplemented with LO (Hong Jing Yuan Lipid Ltd, Ximeng, Inner Mongolia, China), which added at 4% of the concentrate. For two of the dietary treatments, the LO diet was supplemented with dl-malate (LO-M; 99.99%, free acid type; Fu Sang Ltd., Qingdao, China), which added at 2% of the concentrate, or with fumarate (LO-F; 99.99%, free acid type; Fu Sang Ltd., Qingdao, China), which added at 2% of the concentrate. The basal diet was provided as two equal portions of 5.0 kg (DM) at 0800 and 1800 h. Water and trace mineral salts were available free choice.

**Table 2:** Ingredients of the concentrate and nutritional compositions of the diets.

Composition	Diet^1^			
	CON	LO	LO-M	LO-F
Ingredients (% of dry matter (DM))				
Commercial concentrate	80	80	80	80
Rice straw	20	20	20	20
Linseed oil (% of concentrate)		4	4	4
DL-Malate (% of concentrate)			2	
Fumarate (% of concentrate)				2
Chemical composition (% of DM)				
DM	84.53	85.78	85.28	86.03
Crude protein	15.75	15.36	15.28	15.74
Ether extract	4.37	8.12	8.28	8.39
Neutral detergent fiber	28.71	28.65	29.23	29.04
Crude ash	7.31	7.45	7.21	7.56

^1^ CON, steers were fed the basal diet only; LO, steers were fed the CON diet supplemented with linseed oil (4% of diet DM); LO-M, steers were fed the CON diet supplemented with linseed oil (4% of diet DM) and malate (2% of diet DM); LO-F, steers were fed the CON diet supplemented with linseed oil (4% of diet DM) and fumarate (2% of diet DM).

### Sample collection and chemical analysis

The rumen contents were collected on two consecutive days via a rumen fistula 3 h and 6 h after feeding the diets in each experimental period. The rumen contents were strained through four layers of cheesecloth to separate the rumen fluid from particulate matter at each sampling time point. The pH of the rumen fluid was measured immediately and 5 mL of rumen fluid was collected for ammonia-N and VFA analysis. All of the rumen fluid samples were frozen at—20°C until their analysis.

The ammonia-N concentration was determined as described previously [38] using a UV/VIS spectrophotometer (Optizen 3220UV, Mecasys Co. Ltd, Daejeon, Korea). A 0.8-mL aliquot of culture solution was mixed with 0.2 mL 255 phosphoric acid and 0.2 mL 2% pivalic acid solution as an internal standard for VFA analysis. The VFA concentration was determined by gas chromatography (GC7890A, Agilent, Santa Clara, CA, USA), equipped with a flame ionization detector. Oven temperature used for VFA analysis was 120°C and the injector and detector temperatures were maintained at 170°C and 200°C, respectively. A 30-m fused silica capillary column (HP-FFAP, 19091F-112, 0.32 mm i.d.; Agilent) was used. The carrier gas was ultra-high purity helium (flow rate 50 mL/min). VFAs were identified and quantified by comparison to authentic VFA standards (Supelco 47058, WSFA-4, USA). Additionally, 15 mL of rumen fluid was collected from each steer at 3 h and 6 h after feeding, and these samples were stored at—80°C until DNA extraction.

### DNA extraction and purification

Genomic DNA was extracted from each sample using an E.Z.N.A. Soil DNA Kit (Omega Bio-Tek, Inc., Norcross, GA, USA) according to the manufacturer’s instructions. An ND-2000 spectrophotometer (Nanodrop Inc., Wilmington, DE, USA) was used to evaluate DNA quality. To ensure the sample quality, the relative ratio of the absorbance values at 260 and 280 nm was established at 1.8–2.0, and the relative ratio of the absorbance values at 260 and 230 nm was established to be >1.7. Extracted DNA was stored at—20°C.

### PCR amplification and 454 pyrosequencing

An approximately 500-base pair (bp) region in the bacterial 16S rRNA gene that spanned the V1–V3 regions was selected to construct a community library by tag pyrosequencing. The bar-coded, broadly conserved primers 8F-B (5′-AGAGTTTGATCCTGGCTCAG-3′, *Escherichia coli* bases 8 to 27) and 533R-A (5′-TTACCGCGGCTGCTGGCAC-3′, *E*. *coli* bases 1522 to 1504) for rumen bacteria were used to amplify this region, where these primers contained A and B sequencing adaptors (454 Life Sciences, Branford, CT, USA). The PCR reactions were performed in triplicate in a 50-μL mixture, which contained 0.2 μM of each forward and reverse primer, 2 μL of template DNA, 5 μL 10× PCR reaction buffer (Mg^2+^), 0.2 mM dNTP, and 2.5 U of *Pfu* DNA polymerase (MBI Fermentas, Burlington, Ontario). The PCR program for the 16S rDNA of rumen bacteria was: initial denaturation at 94°C for 5 min; 30 cycles of denaturing at 94°C for 30 s,; annealing at 55°C for 30 s; extension at 72°C for 1 min; and a final extension at 72°C for 10 min. Replicate PCR products of the same sample were assembled within a PCR tube. The PCR products were separated on an agarose gel (2% in Tris/borate/EDTA [TBE] buffer) containing ethidium bromide and purified using a DNA gel extraction kit (Axygen, Union City, CA, USA).

The DNA concentration of each PCR product was determined using a Quant-iT PicoGreen double-stranded DNA assay kit (Invitrogen, Carlsbad, CA, USA) prior to sequencing and DNA quality was evaluated using an Agilent 2100 Bioanalyzer (Agilent). Following quantification, the amplicons from each reaction mixture were pooled in equimolar ratios based on their concentration and subjected to emulsion PCR to generate amplicon libraries, as recommended by 454 Life Sciences. Amplicon pyrosequencing was performed from the A-end using a 454/Roche A sequencing primer kit on a Roche Genome Sequencer GS FLX Titanium platform (Majorbio Bio-Pharm Technology Co. Ltd, Shanghai, China).

### Statistical and sequencing analyses

The data were analyzed as a replicated 4 × 4 Latin square using analysis of variance (ANOVA) with the general linear models procedure of SAS [39]. The model included the main effects of diet, sampling time, the diet x sampling time interaction, and steers. Means were separated based on the least significant differences, where the process was protected by the overall *F-*value for diet (*P* < 0.05).

PCA was conducted using R to group the microbial communities present in different samples. Sequences were denoised and analyzed using the Quantitative Insights into Microbial Ecology (QIIME) Software [40]. Valid reads were determined according to the following major principles: check the completeness of barcodes and exclude sequences with even a single base bias; ensure that sequences comprise >450 bp and <1,000 bp; remove bases at the sequence tail with a sequencing quality score <25; and ensure that the sequence quality values are >20 for >80% of the total bases in each sequence. The pyrosequencing reads were simplified using the “unique.seqs” command to generate a unique set of sequences and then aligned using the “align.seqs” command, before comparisons with the Bacterial SILVA database (SILVA version 108; http://www.arb-silva.de/documentation/background/release-108%20/). The sequence data were deposited in NCBI Sequence Read Archive (SRA, http://www.ncbi.nlm.nih.gov/Trace/sra) under accession number SRA139662. The aligned sequences were trimmed further and redundant reads were eliminated using the “screen.seqs”, “filter.seqs”, and “unique.seqs” commands, in this order. The “chimera.slayer” command was used to identify chimeric sequences. The “dist.seqs” command was executed and unique sequences were clustered into OTUs, which were defined as ≥97% similarity. Rarefaction analysis and Good’s coverage were determined for the CON, LO, LO-M, and LO-F libraries. Venn diagrams, and OTU rank abundance distribution curves were generated using custom Perl scripts, and PCA was performed based on the weighted UniFrac distance. Data preprocessing, OTU-based analysis, and hypothesis testing were performed using the mothur software [41].
